# Silica Nanoparticle Internalization Improves Chemotactic Behaviour of Human Mesenchymal Stem Cells Acting on the SDF1α/CXCR4 Axis

**DOI:** 10.3390/biomedicines10020336

**Published:** 2022-02-01

**Authors:** Emanuela Vitale, Daniela Rossin, Sadia Perveen, Ivana Miletto, Marco Lo Iacono, Raffaella Rastaldo, Claudia Giachino

**Affiliations:** 1 Department of Clinical and Biological Sciences, University of Turin, 10043 Orbassano, Italy; emanuela.vitale@unito.it (E.V.); d.rossin@unito.it (D.R.); sadia.perveen@unito.it (S.P.); marco.loiacono@unito.it (M.L.I.); claudia.giachino@unito.it (C.G.); 2 Department of Science and Technological Innovation, University of Eastern Piedmont, 15121 Alessandria, Italy; ivana.miletto@uniupo.it

**Keywords:** human mesenchymal stem cells, regenerative medicine, silica nanoparticles, hMSC homing, SDF1α/CXCR4 axis, tissue injury, autophagy, cell migration, TNFα/TNFRs axis

## Abstract

Human mesenchymal stem cell (hMSC)-based therapy is an emerging resource in regenerative medicine. Despite the innate ability of hMSCs to migrate to sites of injury, homing of infused hMSCs to the target tissue is inefficient. It was shown that silica nanoparticles (SiO_2_-NPs), previously developed to track the stem cells after transplantation, accumulated in lysosomes leading to a transient blockage of the autophagic flux. Since CXCR4 turnover is mainly regulated by autophagy, we tested the effect of SiO_2_-NPs on chemotactic migration of hMSCs along the SDF1α/CXCR4 axis that plays a pivotal role in directing MSC homing to sites of injury. Our results showed that SiO_2_-NP internalization augmented CXCR4 surface levels. We demonstrated that SiO_2_-NP-dependent CXCR4 increase was transient, and it reversed at the same time as lysosomal compartment normalization. Furthermore, the autophagy inhibitor Bafilomycin-A1 reproduced CXCR4 overexpression in control hMSCs confirming the direct effect of the autophagic degradation blockage on CXCR4 expression. Chemotaxis assays showed that SiO_2_-NPs increased hMSC migration toward SDF1α. In contrast, migration improvement was not observed in TNFα/TNFR axis, due to the proteasome-dependent TNFR regulation. Overall, our findings demonstrated that SiO_2_-NP internalization increases the chemotactic behaviour of hMSCs acting on the SDF1α/CXCR4 axis, unmasking a high potential to improve hMSC migration to sites of injury and therapeutic efficacy upon cell injection in vivo.

## 1. Introduction

Mesenchymal stem cells (MSCs) possess the capability for self-renewal, multilineage differentiation, and also display angiogenic and immunomodulatory properties that make them attractive for clinical applications [1,2,3]. Indeed, MSCs are widely used in regenerative medicine in order to promote healing of damaged tissues and organs; as well, they represent a promising cell source for ischaemic disease therapy [1,2]. An important premise of successful MSC-based therapies is that these cells reach the site of injury and home to the damaged area where they can participate in tissue regeneration in response to inflammatory and ischaemic signals [3].

The majority of chemotactic stimuli activate cell surface receptors that belong to the G protein-coupled receptor (GPCR) superfamily, with the stromal cell-derived factor 1α (SDF1α)/C-X-C motif chemokine receptor 4 (CXCR4) axis representing the prototypic signal system. SDF1α, a chemotactic factor encoded by the CXCL12 gene, is critical for stem/progenitor and mesenchymal cell recruitment [4,5,6]. The SDF1α/CXCR4 axis exerts a pivotal role in chemotaxis during embryonic development, while after birth it recruits cells to the sites of injury [5]. Indeed, SDF1α is upregulated in response to tissue injury [7] and acts as a chemoattractant for recruiting circulating or resident MSCs to lesion sites through interaction with its cognate receptor CXCR4 located on the membrane of MSCs [3,6,8,9,10].

Although MSCs are recognized as the best potential candidates for cell therapy, some limitations still exist. In vivo experiments pointed out limited efficacy of MSC-based therapies due to inefficient homing and very low (<2%) cell engraftment rate in the damaged tissue [11], irrespective of the route of delivery (intravenous injection or transplantation). This represents a major bottleneck in achieving the full therapeutic potential of MSC-based therapies. One reason for the inefficient homing of MSCs could be due to the fact that there is very low expression of SDF-1 receptor, CXCR4, on their surface [9,12,13,14]. Moreover, in vitro expansion of MSCs gradually leads to loss in expression of key receptors, particularly CXCR4 [10,15,16], that further reduces the homing potential of engrafted MSCs. Besides this, scarcity of nutrients and oxygen supply owing to insufficient vascularization, together with ischemia-induced oxidative stress further contributes to low retention rate and loss of transplanted MSCs [11,17]. In spite of these factors, MSCs are able to secrete antioxidant molecules, paracrine factors (such as βFGF and VEGF) and extracellular vesicles (containing various molecules among which miRNAs) which can favour vasculogenesis and angiogenesis [1,18], that contribute to attaining a prompt homing of these cells, thus ameliorating their engraftment potential as well.

Nanotechnologies can improve the efficacy of stem cell therapies. First, as imaging agents they allow a direct observation of delivered cells from their injection to their engraftment sites [19,20]. Second, they may act as cargos for gene/drug delivery [21,22,23]. In addition, very recent evidence suggests that NPs can act in a third way by directly affecting stem cell behaviour [24,25]. Keeping this in view, previously we have developed a method of fluorescent staining with silica NPs (SiO_2_-NPs) suited to tracking human MSCs (hMSCs), and we have validated this approach both in vitro [26,27] and ex vivo [28]. We demonstrated that SiO_2_-NP uptake by hMSCs was well tolerated in the long term, did not induce cytotoxicity or genotoxicity, and did not alter the proliferation and differentiation potential of hMSCs [26,27,28]. Moreover, we observed that SiO_2_-NPs directly improved adhesion properties of hMSCs and positively modulated the expression of Connexin-43 [25]. Mechanistically, we demonstrated that, after internalization, SiO_2_-NPs accumulated in lysosomal compartments leading to a transient block of autophagic flux responsible for the increased adhesive phenotype and Connexin-43 expression, being both processes modulated by autophagic activity [25].

Since CXCR4 turnover and its signalling are regulated mainly by the autophagic process [29,30], in this work, we assessed whether SiO_2_-NP uptake would have an effect on chemotactic migration of hMSCs acting on the SDF1α/CXCR4 axis. An approach that induces CXCR4 expression enhancement on hMSCs would be a useful strategy to improve their migration and subsequent engraftment in injured tissues.

## 2. Materials and Methods

### 2.1. Silica Nanoparticle Production

Pure SiO_2_-NPs and red fluorescent cyanine dye-doped SiO_2_-NPs were prepared by a water-in-oil microemulsion, as detailed by Alberto et al. [31]. For the preparation of dye-doped nanoparticles, IRIS3 cyanine (by Pianeta S.r.l., formerly Cyanine Technologies S.p.A., Torino, Italy) was added to the reaction system in the form of aminopropyltriethoxysilane derivative. As typical for trimethine cyanine dyes, the absorption and photoluminescence spectra of IRIS 3 range from ca. 450 to 575 nm and from 500 to 700 nm, respectively. At the end of the process the cyanine molecules resulted were stably entrapped in the bulk of NPs based on solvatochromism test, thus the surface chemistry of NPs was not affected by the presence of IRIS3 molecules. Obtained SiO_2_-NPs (both pure and dye-doped) exhibited a diameter of 50 ± 2 nm and possessed elevated morphologic homogeneity, as well as good colloidal stability (Figure S1). When containing cyanine molecules, they displayed bright fluorescence emission and high photostability [31,32]. SiO_2_-NPs were almost monodispersed in water; in complete DMEM 1% FBS, SiO_2_-NPs resulted in the formation of agglomerates of ca. 1 μm in size [25]. Ultrasonic sonication was performed prior to cell treatment.

### 2.2. Culture and Cell Treatments

The hMSCs isolated from the bone marrow of healthy donors were commercially obtained from ATCC. For all the experiments cells between passages 4 to 8 were used. hMSCs were cultured in DMEM supplemented with 1% sodium pyruvate, 1% nonessential amino acids, 1% kanamycin, 1% L-glutamine, 0.1% β-mercaptoethanol (complete DMEM) and 10% foetal bovine serum (FBS) (standard medium) (all from Sigma Aldrich S.r.l., Milan, Italy) and kept in an atmosphere of 5% CO_2_, 95% air at 37 °C in a humidified incubator. Cells were expanded at a seeding density of 3500 cell/cm^2^ and subcultured twice a week. Exponentially growing hMSCs were seeded at 6500 cell/cm^2^ 24 h before the two following protocols: (a) SiO_2_-NPs: cells were exposed for 16 h to a suspension of water dissolved dye-doped SiO_2_-NPs 50 μg/mL in complete DMEM 1% FBS; for some in vitro experiments, utilized SiO_2_-NPs contained no fluorophore to avoid any possible interference of fluorescence spectra; (b) Control (CTR): cells were incubated for 16 h in complete DMEM 1% FBS supplemented with the same volume of sterile H_2_O in which SiO_2_-NPs were dispersed. Subsequently, samples of both conditions were washed twice with warm phosphate-buffered saline (PBS) and analysed immediately after treatment (Day 0) or after 1, 4 and 8 days of in vitro recovery in standard medium; brightfield microscopy images showing hMSCs under treatment with SiO_2_-NPs are available in the supplementary data (Figure S2). For inhibition of lysosomal degradation activity hMSCs were starved for 2 h with DMEM 0% FBS and then treated using 100 nM Bafilomycin A1 for 2 h. Human umbilical vein endothelial cells (HUVECs) cells were isolated from donors and cultured in EGM-2 media in an atmosphere of 5% CO_2_, 95% air at 37 °C in a humidified incubator. For hypoxic experiments, cells were cultured in a hypoxic chamber with 1% O_2_ and 5% CO_2_ conditions for 6 h. Cells were serum-starved in EBM-2 plus 0.5% FBS at least 6 h before hypoxic culture to minimize the effects of growth factors in the expansion media.

### 2.3. Cell Migration Assay

#### 2.3.1. Transwell Assay

hMSCs were seeded at a density of 7 × 10^3^ cells/cm^2^ followed by treatment with either CTR or SiO_2_-NPs protocols for 16 h. Afterwards cells were detached with trypsin and seeded on polyethylene terephthalate (PET) membranes of 24-well BD Falcon culture inserts (6.5 mm diameter of insert, 8.0 μm pore size of membrane) at the final density of 5 × 10^4^ cell/cm^2^ in DMEM supplemented with 0.5% FBS. Fibronectin (Sigma Aldrich S.r.l.) 50 µg/mL coating on membranes was performed by incubating the inserts for 2 h at 37 °C in a humidified 5% CO_2_ atmosphere. Inserts were placed in 24 well plates, and the lower chambers of 24-well systems were filled with 600 μL of either DMEM 0.5% FBS (negative), DMEM 0.5% FBS with SDF1α (Miltenyi Biotec, Bologna, Italy) 100 µg/mL or tumour necrosis factor α (TNFα) (Miltenyi Biotec) 10 ng/mL, conditioned media (EBM plus 0.5% FBS) from HUVECs cultured in different oxygen tensions and DMEM 30% FBS (positive). The multiwells containing the cell culture inserts were incubated at 37 °C in a humidified 5% CO_2_ atmosphere for 24 h. Afterwards, culture inserts were rinsed with PBS and cells attached to the upper side of the membrane were mechanically removed by a cotton-tipped applicator. Cells that migrated to the lower side of the membrane were fixed by treatment with 2.5% glutaraldehyde for 30 min at RT and stained with 2% crystal violet for 1 h at RT. For each insert, five random fields were acquired with an inverted microscope Motic AE 2000. Finally, the cells were counted by ImageJ^®^ software (Available online on http://rsb.info.nih.gov/ij/; accessed on 30 November 2021) and the total number of migrated cells per membrane was calculated.

#### 2.3.2. µ-Slide Chemotaxis Chamber Assay

Cell migration assays were performed using the μ-Slide Chemotaxis (© ibidi, GmbH, Martinsried, Germany) according to the manufacturer’s protocol. The hMSCs were seeded at a density of 7 × 10^3^ cells/cm^2^ followed by treatment with either CTR or SiO_2_-NPs protocols for 16 h. Afterwards cells were detached with trypsin and 12 × 10^3^ hMSCs were seeded in the central canal and incubated for 2 h at 37 °C in a humidified 5% CO_2_ to allow cell attachment. The directional gradient was created filling the first reservoir with FBS at 0.5% and the second with FBS at 0.5% and SDF1α (Miltenyi Biotec) 100 µg/mL. Live cells imaging was performed for 60 h with Leica SP5 inverted confocal time-lapse microscope in which cells remain alive at 37 °C and 5% CO_2_ conditions. Images were analysed with ImageJ Manual Tracking Plugin and Chemotaxis and Migration Tool.

### 2.4. Confocal Microscopy

Adhered hMSCs at the end of experiments were processed for confocal immunofluorescence. Cells were fixed with 4% PFA, permeabilized with 1% Triton X-100 and blocked with 6% (*w/vol*) BSA and 2.5% (*vol/vol*) normal goat serum. For CXCR4 analysis cells were stained over night at 4 °C with the primary antibody mouse anti-CXCR4 (Abcam, Milan, Italy) (1:50) and then incubated for 30 min at room temperature with the secondary antibody goat anti-mouse Alexa Fluor 488 (Thermo Fisher Scientific Inc., Monza, Italy) (1:500). Quantification was assessed using ImageJ^®^. To mark late endosomes and lysosomes, cells were incubated for 15 min at 37 °C with LysoTracker Green (2 μmol/L) (Thermo Fisher Scientific Inc.) and analyzed with inverted confocal laser scanning microscope LSM 800 (Carl Zeiss Inc., Oberkochen, Germany). Nuclear staining was performed with Hoechst-33342 (5 μg/mL).

### 2.5. Flow Cytometry

For CXCR4 and TNF receptor (TNFR) surface expression, hMSCs were washed with PBS, blocked with 5% FBS for 30 min at room temperature and stained for 30 min at 4 °C with anti-CXCR4 and anti-TNFR directly conjugated antibody APC (Miltenyi Biotec) (1:50). Cells were acquired with CyAN ADP flow cytometer (Beckman Coulter, s.r.l. Milan, Italy and analysed by software FlowJo^®^ (Available online on https://www.flowjo.com/; accessed on line 30 June 2021). At least 50,000 events per sample were collected.

### 2.6. Western Blot Analysis

hMSCs were lysed with RIPA buffer and 12 μg of proteins were loaded. SDS-PAGE (12% Bis-Tris gel, from Invitrogen, Thermo Fisher Scientific Inc.), Polyvinylidene difluoride transfer membrane and quantification BCA assay (Thermo Fisher Scientific Inc.) were used. Membranes were blocked in 5% milk for 1 h at room temperature and probed with the primary antibodies (anti-LC3B, anti-p62/SQSTM1 purchased from Sigma-Aldrich S.r.l.) (1:1000) overnight at 4 °C, followed by secondary antibodies (1:5000) for 1 h at RT. Bands were visualized using an enhanced chemiluminescence kit (SuperSignal™ West Pico PLUS, from Thermo Fisher Scientific Inc.). Quantification was assessed using Image Lab Software (Bio-Rad Laboratories S.r.l., Segrate, Italy).

### 2.7. Statistical Analysis

Data were expressed as a mean ± standard error of the mean (SEM) of at least three different experiments or as the mean ± standard deviation (SD) of representative experiments. Statistical comparisons were performed with unpaired Student’s *t*-test. Differences with *p* < 0.05 were regarded as statistically significant.

## 3. Results

### 3.1. SiO_2_-NP Internalization Increases Expression of CXCR4 in hMSCs by Inducing a Transient Block of Lysosomal Degradation

**Figure 1:**
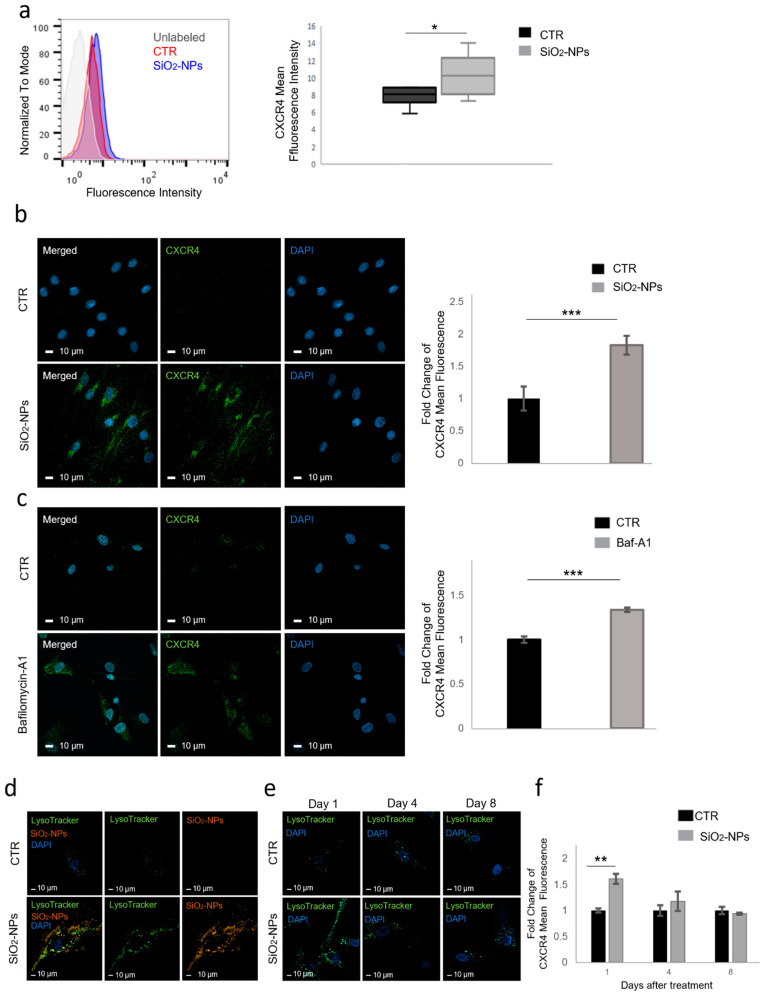
SiO_2_-NP internalization increases CXCR4 expression in hMSCs. (**a**) Fifty thousand of both living untreated (CTR) and SiO_2_-NP-treated hMSCs were incubated with anti-CXCR4 antibody and fluorescence intensity was measured by flow cytometry after 16 h of SiO_2_-NP treatment. Data shown are cytometric profiles of a representative sample from each experimental group: background signal of cells without antibody incubation (grey), control cells (red), SiO_2_-NP-treated cells (blue). Representative histogram of flow cytometry analysis was obtained from FlowJo software. Box plot shows the fluorescence intensity expressed as mean ± SD. (**b**) Representative images of CTR and SiO_2_-NP-treated hMSCs. Cells fixed on coverslips were stained with anti-CXCR4 primary antibody, followed by 488-conjugated (Green) secondary anti-rabbit antibody, and then analysed by confocal microscopy. Nuclei were stained with DAPI (Blue). Magnification 63×; scale bar 10 μm. Quantitative evaluation of fluorescence intensity was expressed as fold changes of SiO_2_-NP-treated hMSCs versus untreated cells. At least 700 cells were analysed for each group. Bars show mean ± SEM. (**c**) Representative images of hMSCs treated with Bafilomycin-A1 (100 nM) and CTR. Cells fixed on coverslips were stained with anti-CXCR4 primary antibody, followed by 488-conjugated (Green) secondary anti-rabbit antibody, and then analysed by confocal microscopy. Nuclei were stained with DAPI (Blue). Magnification 63×; scale bar 10 μm. Quantitative evaluation of fluorescence intensity was expressed as fold changes of SiO_2_-NP-treated hMSCs versus CTR cells. At least 350 cells were analysed for each group. Bars show mean ± SEM. (**d**) Representative confocal images of control or SiO_2_-NP-treated (red) hMSCs stained with LysoTracker (green) and DAPI (Blue). Magnification 63x; scale bar 10 μm. (**e**) Representative images of CTR and SiO_2_-NP-treated hMSCs. Cells fixed on coverslips were labelled with Lysotracker (green) and DAPI (Blue) at different time points (days 1, 4 and 8) and acquired by confocal microscopy. Magnification 63×; scale bar 10 μm. (**f**) CTR and SiO_2_-NP-treated hMSCs were allowed to recover for 1, 4, and 8 days after treatment and then CXCR4 expression was evaluated by flow cytometry. Histogram shows the fluorescence intensity expressed as fold changes of SiO_2_-NP-treated hMSCs versus untreated cells. Bars show mean ± SEM. * *p* < 0.05; ** *p* < 0.01; *** *p* < 0.001; *n* = 3.

CXCR4 fluorescence intensity was measured by flow cytometry in NP-treated and untreated hMSCs, and data showed that SiO_2_-NP internalization significantly increased CXCR4 cell surface expression (*p* < 0.05) compared to control cells after 16 h of SiO_2_-NP treatment (Figure 1a). We then evaluated CXCR4 total expression by immunofluorescence and confocal microscopy analysis. Qualitative observation of images strongly suggested that, after SiO_2_-NP internalization hMSCs increased CXCR4 expression and, indeed, quantitative analysis of fluorescence showed a significant (*p* < 0.001) increase in CXCR4 expression in SiO_2_-NP-treated hMSCs compared to control cells (Figure 1b).

Bafilomycin-A1 is a drug widely used in in vitro studies as it blocks the activity of the autophagic flux via inhibition of the autophagosome–lysosome fusion [33] a mechanism similar to that described for SiO_2_-NPs [25,34,35]. We thus investigated whether Bafilomycin-A1 treatment could induce the same phenotypes observed in SiO_2_-NP-treated hMSCs, as this would confirm the hypothesized mechanism of action. Bafilomycin-A1 was able to block the autophagic flux in control hMSCs, as showed by the increase of LC3II/LC3I ratio accompanied by increased p62 expression (Figure S3). Bafilomycin-A1, instead, did not induce any additive effect on SiO_2_-NP-treated cells, whose autophagic flux was already compromised by the accumulation of nanoparticles in lysosomes (Figure S3). These data support our hypothesis suggesting a common mechanism for both Bafilomycin-A1 and SiO_2_-NPs accumulation.

We then evaluated CXCR4 total expression on Bafilomycin-A1 treated hMSCs by immunofluorescence and confocal microscopy analysis. Qualitative observation of images indicated that the pharmacological blockade of autophagy flux increased CXCR4 expression (Figure 1c, left panels) and quantitative analysis confirmed a significant (*p* < 0.001) increase in cells treated with Bafilomycin-A1 compared to control cells (Figure 1c, right graph).

In agreement with our previously published results [25,28], we observed that in NP-treated hMSCs there is strong colocalization between SiO_2_-NPs and lysosomes. Furthermore, control cells were characterized by the presence of a low number of small-sized lysosomes while hMSCs treated with SiO_2_-NPs exhibited many larger lysosomes (Figure 1d).

The time course analysis of LysoTracker experiments in SiO_2_NP-treated hMSCs indicated that on day 1 accumulation of larger organelles was still present, and this phenomenon began to attenuate on day 4 (Figure 1e, days 1 and 4). On day 8 confocal analysis displayed normalized lysosomal compartment, confirming that SiO_2_-NPs transiently disrupt the lysosomal compartment, and that lysosomal compartment perturbation spontaneously recovers within 8 days from SiO_2_-NP treatment (Figure 1e, day 8).

To investigate the involvement of SiO_2_-NP-mediated autophagic flux blockade in the observed functional effects, we evaluated whether CXCR4 increases in SiO_2_-NP-treated hMSCs followed a similar kinetics. CXCR4 fluorescence intensity was measured by flow cytometry after 1, 4 and 8 days during which hMSCs remained in complete 10% FBS standard medium. Results highlighted that the enhanced CXCR4 expression was still evident on day 1 (*p* < 0.01) (Figure 1f), while it progressively returned to control levels when lysosomal compartment normalization occurs (Figure 1f, days 4 and 8).

These results indicated that (1) SiO_2_-NP internalization increased surface expression of CXCR4 in hMSCs, (2) pharmacological inhibition of lysosomal degradation activity obtained using Bafilomycin-A1, reproduced in hMSCs the same effect as NPs, and (3) the augmented CXCR4 expression phenotype observed in SiO_2_-NP-treated hMSCs was released within 8 days coincident with the lysosomal compartment normalization, thus confirming a transient block of lysosomal degradation as the most possible mechanism of action.

### 3.2. Internalization of SiO_2_-NPs Increases hMSC Migration Specifically in Response to SDF1α

**Figure 2:**
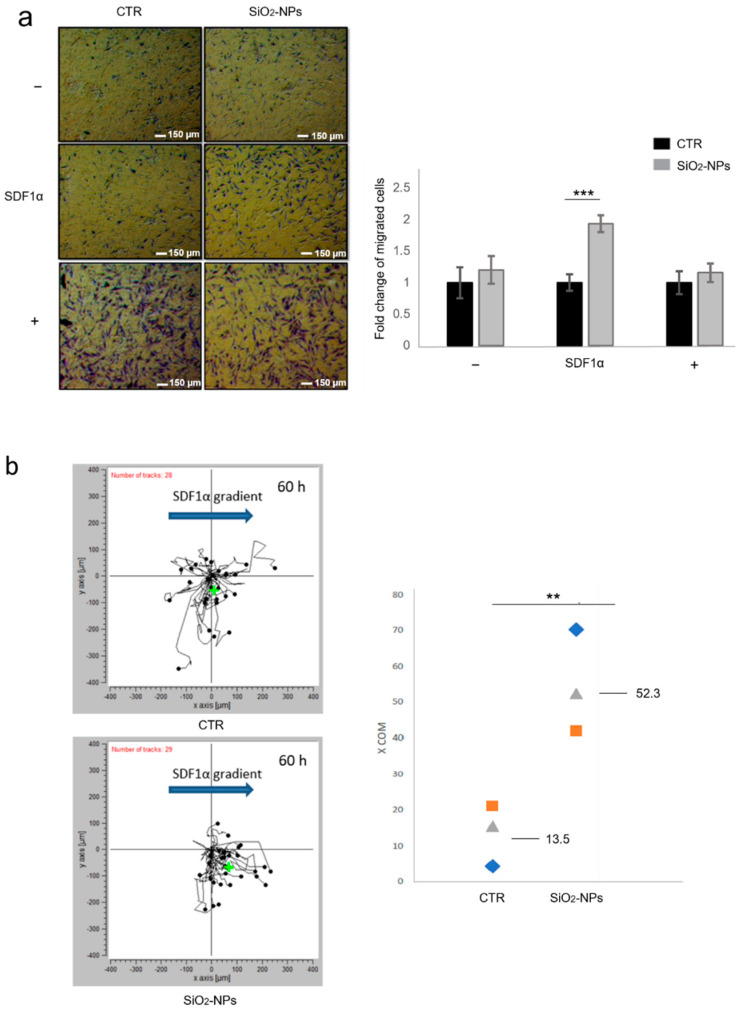
SiO_2_-NP internalization augments hMSC migration in response to SDF1α gradient. (**a**) Representative images of migration of untreated (CTR) and SiO_2_-NP-treated hMSCs in the presence of chemoattractant SDF1α (100 µg/mL) or medium with either 0.5% FBS (negative control; −) or 30% FBS (positive control; +). Once fixed, hMSCs were stained with crystal violet. Magnification 4×; scale bar 150 µm. Bar graphs represent the quantification of migrated cells normalized to untreated experimental group and expressed as fold change of migrated cells. Bars show mean ± SEM. (**b**) Left panel: 2D trajectory plot of hMSCs in a representative experiment showing paths of 28 control cells and 29 SiO_2_-NP-treated cells under 60 h SDF1α directional gradient indicated by the blue arrow. Accumulated distance of cells is reported in a two-axis plot in which distances are expressed in µm. Center of the axis is the starting point of all cells (starting center of mass), while black spots indicate the position of cells at the end of the migration time (endpoints). Green cross is the center of mass at the end of the experiment (60 h), which represents the spatial averaged point of all cell migration endpoints. (**b**) Right panel: COM values of SiO_2_-NP-treated hMSCs compared to control cells. Geometric figures represent COM value of three different experiments, for both control and treated conditions, and on the right the corresponding mean of COM value is reported. ** *p* < 0.01; *** *p* < 0.001. *n* = 3.

Since cell recruitment at sites of injury is a key aspect of MSC-based therapy effectiveness, based on our observation that CXCR4 expression was increased in SiO_2_-NP-treated cells we further evaluated whether the chemotactic behaviour in response to SDF1α gradient was modified by SiO_2_-NP internalization. For this purpose, we performed both tridimensional and bidimensional time-lapse chemotaxis assays. For the tridimensional transwell migration assay, untreated and SiO_2_-NP-treated hMSCs were seeded on the top wells of chemotaxis chambers in the presence of 0.5% FBS. Migration assay was performed in different conditions: migration towards the lower chamber containing 0.5% FBS (negative control; −), migration towards the lower chamber containing the relevant chemoattractant (SDF1α, 100 µg/mL), and migration towards the lower chamber containing 30% FBS (positive control; +). After 24 h, the migrating cells were counted by ImageJ^®^ software, thus calculating total number of migrated cells per membrane. Both qualitative and quantitative observations proved that SiO_2_-NPs enhanced chemotactic behaviour of hMSCs in response to SDF1α in a statistically significant manner (*p* < 0.001) compared to the untreated cells (Figure 2a). It was noted that there was no migration difference between the control cells and the cells treated with nanoparticles towards 30% FBS, suggesting that SiO_2_-NPs did not modify the migratory behaviour of the cells in a generic way towards growth factors, but increased the migration specifically towards SDF1α (Figure 2a).

To further investigate how SiO_2_-NP internalization enhanced migratory capability of hMSCs in response to directional gradient of SDF1α, we used µ-Slide Chemotaxis Chambers (© ibidi GmbH) in conjunction with time-lapse microscopy (Figure 1b). This powerful tool allows us to follow the chemotactic cell response to a chemoattractant gradient in real-time and to obtain a detailed analysis of specific migration behaviour from random movement, thus providing more reliable and accurate results. To perform the experiments, untreated and SiO_2_-NP-treated hMSCs were seeded in the central canal of a chemotaxis chamber for 1 h to allow cell adhesion, then directional gradient was created filling the first reservoir with FBS at 0.5% and the second one with FBS at 0.5% plus SDF1α 100 µg/mL. Cell migration was followed through time-lapse microscopy for 60 h (Figure S4). We analysed the center of mass (COM), a strong parameter for evaluating chemotaxis as it represents the spatial averaged point of all cell endpoints. Depending on the direction in which the population of cells has drifted, COM coordinates can be either positive or negative. The difference in the center of mass, at the beginning and end of the experiment, is called the displacement (or length) of the center of mass. This value represents the length of migration for all the cells. In the absence of chemotaxis, the COM coordinates are not significantly different (0, 0), while strong chemotaxis effects are characterized by a significant COM displacement. We analysed COM values at 60 h time point and we highlighted a greater shift of the center of mass (represented with the symbol +) toward SDF1α gradient in SiO_2_-NP-treated hMSCs (Figure 2b, left panel), confirming that the internalization of SiO_2_-NPs enhanced chemotactic migration of cells in response to SDF1α. Migration was evaluated in three independent experiments and the resulting COM values were represented by the geometric figures proving that SiO_2_-NPs enhance the displacement of the COM from a mean value of 13.4 in control cells to 52.3 in SiO_2_-NP-treated hMSCs (Figure 2b, right panel). In a typical chemotaxis experiment, the COM displacement is time dependent, thus the longer the experimental time, the bigger the displacement of the center of mass. This was indeed confirmed in our experiment, where a time-dependent increase in the COM was evidenced over time (Figure S4, Videos V1 and V2).

**Figure 3:**
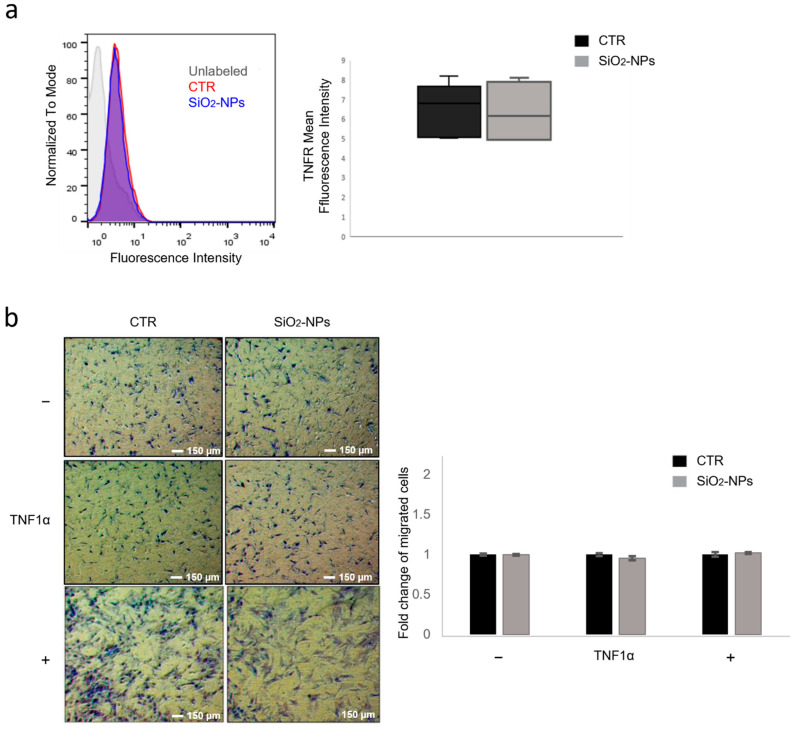
SiO_2_-NPs do not modify either TNFR surface expression or migration toward TNF1α in hMSCs. (**a**) Fifty thousand of both untreated (CTR) and SiO_2_-NP-treated hMSCs were incubated with anti-TNFR antibody and fluorescence intensity was measured by flow cytometry after 16 h of SiO_2_-NP treatment. Data shown are cytometric profiles of a representative sample of each experimental group: background signal of cells without antibody incubation (grey), control cells (red), SiO_2_-NP-treated cells (blue). Box plot shows the mean fluorescence intensity expressed as mean ± SD. (**b**) Representative images of migration of both CTR and SiO_2_-NP-treated hMSCs in the presence of chemoattractant TNFα (10 ng/mL) or medium with either 0.5% FBS (negative control; −) or 30% FBS (positive control; +). Once fixed, hMSCs were stained with crystal violet. Magnification 4×; scale bar 150 µm. Bar graphs represent the quantification of migrated cells normalized to CTR experimental group and expressed as fold change of migrated cells. Bars show mean ± SEM. *n* = 3.

Our hypothesis suggests that only chemotactic receptors whose turnover and activity are mainly regulated by autophagy, such as CXCR4, would show changes upon SiO_2_-NP internalization by hMSCs. For this reason, we studied the cell surface expression of TNFR that is degraded by the proteasome, and the TNFα/TNFR chemotactic axis to understand whether enhanced migration was peculiar of the SDF1α/CXCR4 axis or could include other chemotactic axis indiscriminately. To evaluate whether SiO_2_-NPs could change TNFR expression on hMSC surface, we measured TNFR fluorescence intensity by flow cytometry in control and treated cells. In accordance with our hypothesis, the results showed that SiO_2_-NP internalization did not increase TNFR expression (Figure 3a). To analyse TNFα/TNFRs chemotactic axis in hMSCs, control and SiO_2_-NP-treated hMSCs were seeded on the top well of a chemotaxis chamber and transwell migration assay was carried out on cells migrating towards 3 different conditions: the lower chamber containing medium with 0.5% FBS (−), containing the chemoattractant TNFα (10 ng/mL) or containing 30% FBS (+). After 16 h both qualitative and quantitative analyses highlighted that SiO_2_-NP internalization did not change the migratory behaviour toward TNFα compared to control cells (Figure 3b).

### 3.3. SiO_2_-NPs Augment hMSC Migration in Response to Hypoxia-Induced SDF1α in HUVECs

Hypoxia is a fundamental mechanism governing recruitment and retention of stem and progenitor cells and indeed, hypoxic microenvironments (such as injured tissue) facilitate cell recruitment and retention in ischemic tissues [36,37].

**Figure 4:**
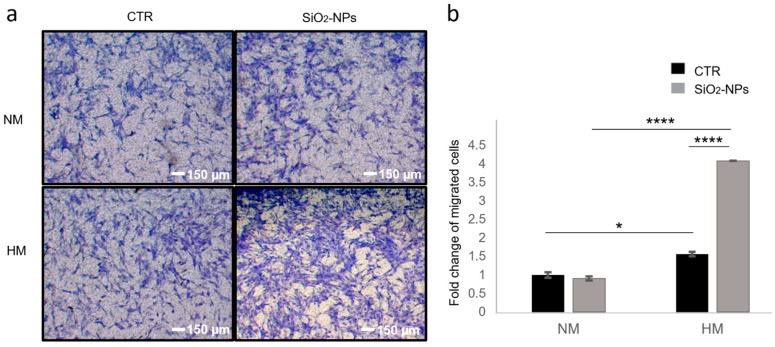
SiO_2_-NP internalization promotes hMSC migration in response to hypoxia-induced SDF1 in HUVECs. (**a**) Representative images of migration of untreated (CTR) and SiO_2_-NP-treated hMSCs to conditioned media from HUVECs cultured in hypoxic conditions (HM) or media from HUVECs grown in normal oxygen tension (NM). Once fixed, hMSCs were stained with crystal violet. Magnification 4×; scale bar 150 µm. (**b**) Bar graphs represent the quantification of migrated cells normalized to the NM migrating CTR experimental group and expressed as fold change of migrated cells. Bars show mean ± SEM. * *p* < 0.05; **** *p* < 0.0001. *n* = 3.

Hence, in order to reproduce in vitro a condition that occurs in vivo after an ischaemic event, in which cells exposed to hypoxic stress release SDF1α to recruit progenitor and stem cells to the damage area, we used HUVECs. These cells produce SDF1α when undergoing hypoxia [38] and we investigated whether SiO_2_-NP-treated hMSCs migrated more efficiently towards hypoxia-induced SDF1α compared to control cells. For this purpose, we performed a transwell assay seeding on the top well of a chemotaxis chamber either SiO_2_-NP-treated hMSCs or control cells, whereas the lower chamber was filled with either conditioned media from HUVECs cultured in hypoxic conditions (HM) or media from HUVECs grown in normal oxygen tension (NM). After 24 h, the migrated cells were counted calculating the total number of migrated cells per membrane. As expected, cells migrating towards NM did not show any migration differences (Figure 4). However, both qualitative and quantitative analyses demonstrated that control cells increase their migration with a significant statistical difference (*p* < 0.05) in response to HM since hMSCs expressing CXCR4 are competent to migrate toward SDF1α released from hypoxic culture HUVECs (Figure 4). Importantly, the migration of hMSCs was further increased (*p* < 0.0001) after treatment with SiO_2_-NPs, underlining the efficacy of nanoparticles to increase the recruitment of hMSCs in damaged tissues subjected to hypoxic stress (Figure 4).

## 4. Discussion

Based on our previous published results, that highlighted a transient block of autophagic flux mediated by SiO_2_-NP internalization [25], in this work we hypothesized that SiO_2_-NPs would have effect on the chemotactic migration of hMSCs acting on the SDF1α/CXCR4 axis, since CXCR4 turnover and signalling are regulated mainly by the autophagic process [29,30]. Here, for the first time, we have demonstrated an increased expression of CXCR4 and an improved migratory capability of hMSCs toward synthetic and natural SDF1α mediated by silica nanoparticles.

The importance of this finding derives from the observation that despite the innate migration capacity of hMSCs; unfortunately, the homing of MSCs is inefficient, with only a small percentage of cells reaching the target tissue after systemic administration and consequently in the clinical studies MSCs have shown limited therapeutic benefit [11,39]. Many strategies have been employed in the hope of improving this process [3]; specifically, the increase of CXCR4 in MSCs was obtained through genetic modification [18,40,41,42,43], cell surface engineering [44], in vitro priming of MSCs [45,46,47,48,49] and ultrasound techniques [50]. Interestingly, iron oxide-based NPs have also demonstrated an impact on MSC chemotaxis, yet through radically different mechanisms of action involving iron ions release from NPs after cellular uptake, which in turn enhanced hypoxia-inducible factor-1 (HIF-1) and CXCR4 expression [51,52]; however, full details on how iron oxide NPs can impact chemotaxis in MSCs remain to be fully elucidated.

Furthermore, we observed a similar mechanism between SiO_2_-NP accumulation and Bafilomycin-A1 treatment that leads to CXCR4 overexpression, confirming the direct effect of the autophagic degradation blockage on CXCR4. Indeed, autophagy inhibition has a great impact on chemotactic cell migration and the functional connection between these two processes is exemplified by the action of CXCR4 itself on the autophagy machinery [53]. Upon ligand-binding, CXCR4 triggers a marked reduction in the biogenesis of autophagosomes impairing recruitment of ATG16L1, a component of LC3 conjugation system that mediates the elongation and closure of the phagophore, to the initial endocytic vesicles; in this way, CXCR4 blocks the autophagic flux in the initial step of the process [54]. This anti-autophagic action of CXCR4 is crucial for maintenance of correct balance between assembly and disassembly of adhesion complexes at cell front required for chemotaxis. Coly et al. reported that in both HEK-293 and U87 glioblastoma cells the impact on autophagy induced by GPCRs is confined at the front of migrating cells, where the receptor is activated, as it required a local action to favour the efficient formation of adhesions, while in the rest of the cell the autophagic process remains active [55]. Mechanistically, we propose that the autophagic block produced by SiO_2_-NP internalization renders hMSCs more prone to perform chemotaxis, working in combination with the autophagic inhibition induced by CXCR4 upon ligand binding, contributing to generate the observed effect. Further, we suggest that SiO_2_-NPs improve chemotaxis by both augmenting CXCR4 surface expression and optimizing lamellipodial adhesions, propaedeutic for chemotaxis, in our cell model. This would be coherent with our previous findings [25] demonstrating a largely increased hMSC adhesion ability following treatment with SiO_2_-NPs.

We showed that after SiO_2_-NP treatment the increase of CXCR4 in the membrane is a swift and transient effect. In fact, as in hMSCs the block of the autophagic flux is released within 8 days from the treatment with SiO_2_-NPs, the expression of CXCR4 returns to the control levels in the same time interval. On one hand, these validated the hypothesis that the effects produced by the treatment with nanoparticles on the increase of CXCR4 can be explained by the impact that the accumulation of SiO_2_-NPs in lysosomes has on autophagy. On the other hand, they evidenced a kinetics of SiO2-NP-induced CXCR4 overexpression that is indeed clinically relevant, as predicted by a physiologically-based pharmacokinetic model for the in vivo kinetics of MSCs characterizing and predicting the organ distribution of administered MSCs [56]. Furthermore, CXCR4 kinetics has also been predicted by a more recently developed mathematical model that considers both the kinetics of MSCs and SDF1 in blood and organs, assuming that MSCs arrest in organs via both passive homing through blood flow and active homing through the organ-released SDF1 attracting CXCR4-expressing MSCs [57]. In both models, 1–3 days was the crucial time within which efficient homing takes place, which coincides with the SiO_2_-NP-induced overexpression of CXCR4 that we observed in the hMSC experiments. Further, in vivo studies pointed out that while a transient enhancement of SDF1 level occurred immediately after an ischaemic episode, the upregulation of CXCR4 on bone marrow and resident stem cells started to increase only 4 days after this event when the chemotactic factor was almost absent [58,59,60]. The limited tissue regeneration was attributed to this temporal discrepancy, which might be overcome by early induction of CXCR4 expression, achievable through the SiO_2_-NP-induced overexpression of CXCR4 in hMSCs.

It should be noted that our migration experiments proved that SiO_2_-NP internalization increases hMSC migration in response to SDF1α gradient but did not display any migration difference between the control cells and the cells treated with nanoparticles towards 30% FBS used as positive control. These results suggest that SiO_2_-NPs do not modify the migratory behaviour of the cells generically towards growth factors, but they increase the migration specifically towards SDF1α.

On the basis of our working hypothesis, SiO_2_-NPs inducing a block of autophagic flux would cause effects only on receptors whose turnover is regulated by autophagy such as CXCR4. To confirm this hypothesis, we studied TNFR that is degraded by the proteasome and the TNFα/TNFRs chemotactic axis to understand whether enhanced migration was peculiar for SDF1α/CXCR4 axis or could include another chemotactic axis indiscriminately. Unlike CXCR4, there is no evidence in the scientific literature of a direct involvement of autophagy in either the turnover or the activity of TNFRs, but instead, TNFR signalling is modulated by proteasomal activity [61]. In agreement with our hypothesis, we showed that treatment with SiO_2_-NPs did not modify the expression of TNFR in the membrane and did not induce any effect on cell migration towards TNFα. These data confirmed that the effects on the chemotactic phenotype in hMSCs treated with SiO_2_-NPs are specific for those chemotactic axes that are directly modulated by autophagy.

One common element in the environments of injured or ischemic tissue is the presence of a hypoxic stimulus. SDF1 and its receptor CXCR4 are critical mediators for the ischemia-specific recruitment of circulating progenitor cells. The endothelial expression of SDF1 acts as a signal indicating the presence of tissue ischemia, and its expression is directly regulated by HIF-1 [7,62]. Interestingly, both SDF1 and hypoxia are present in the bone marrow niche, suggesting that hypoxia may be a fundamental requirement for progenitor cell trafficking and function. As such, ischemic tissue may represent a conditional stem cell niche, with recruitment and retention of circulating progenitors regulated by hypoxia through differential expression of SDF1.

Given these premises, to test the effects of nanoparticles in hMSCs in a condition of hypoxic stimulus, we generated in vitro a hypoxic condition typical of environments of injured or ischemic tissue. We used HUVECs that produce SDF1α when subjected to hypoxia [38]. Ceradini and colleagues showed in fact that SDF1 gene expression is regulated by the transcription factor HIF-1 in endothelial cells, resulting in selective in vivo expression of SDF1 in ischemic tissue in direct proportion to reduced oxygen tension [38]. The HIF-1-induced SDF1 expression increased the adhesion, migration and homing of circulating CXCR4-positive progenitor cells to ischemic tissue and the blockade of SDF1 in ischemic tissue or CXCR4 on circulating cells prevented progenitor cell recruitment to sites of injury [38]. We observed that, irrespectively from SiO_2_-NP internalization, cells migrating to media from HUVECs cultured in normoxia were very few. Instead, control cells increased their migration in response to media from HUVECs cultured in hypoxia since hMSCs expressing CXCR4 are competent to migrate to SDF1α released from HUVECs cultured in hypoxic condition. Importantly, we highlighted that the migration of hMSCs was further increased after treatment with SiO_2_-NPs, underlining the efficacy of nanoparticles to increase the recruitment of hMSCs in damaged tissues subjected to hypoxic stress.

The optimal biosafety profile of SiO_2_-NPs previously demonstrated in hMSCs along with preservation of their stemness, proliferation and differentiation properties [28] allows one to include the clinical application of SiO_2_-NP treated hMSCs among future prospects. The first goal would be to test the therapeutic efficacy of hMSCs treated with SiO_2_-NPs in vivo in a preclinical model of induced myocardial infarction. Indeed, we have already demonstrated ex vivo in a beating heart model subjected to ischemia the ability of SiO_2_-NPs to increase hMSC engraftment as well as to improve formation of functional gap junctions with consequent improvement of intercellular communication between hMSCs and H9C2 cells [25]. Considering the modified microenvironment due to a myocardial infarction, in which cells exposed to hypoxic stress will release SDF1α, the augmented CXCR4 expression demonstrated in this work would increase the homing of cells treated with SiO_2_-NPs towards the injured area, potentially improving the therapeutic efficacy of the transplanted cells.

## 5. Conclusions

In conclusion, our results demonstrate that SiO_2_-NP internalization increases CXCR4 expression and the migratory capacity of hMSCs towards SDF1α, unmasking a high potential to improve the homing of hMSCs to the lesion sites. Interestingly, we proved that treatment with SiO_2_-NPs may be a suitable method to functionalize hMSCs without the need to use other approaches that involve cell manipulation or specific culture conditions, improving the therapeutic efficacy of hMSCs after cell injection in vivo while, at the same time, allowing efficient fluorescent tracking of injected cell distribution.

## Data Availability

Not applicable.

## Supplementary Materials

The following are available online at https://www.mdpi.com/article/10.3390/biomedicines10020336/s1, Figure S1: Production of SiO_2_-NPs, Figure S2: Bright field microscopy of SiO_2_-NP-treated hMSCs, Figure S3: Autophagy marker analysis, Figure S4: Trajectory plot of control and SiO_2_-NP-treated hMSCs, Video V1: Trajectory video of progressive migration until 60 h of control hMSCs in response to SDF1α gradient, Video V2: Trajectory video of progressive migration until 60 h of SiO_2_-NP-treated hMSCs in response to SDF1α gradient.
