# Supplementary material for: Silica Nanoparticle Internalization Improves Chemotactic Behaviour of Human Mesenchymal Stem Cells Acting on the SDF1α/CXCR4 Axis
Source: Biomedicines. 2022 Feb 1;10(2):336. doi: 10.3390/biomedicines10020336 (PMC8961775; doi:10.3390/biomedicines10020336)
Supplement: Supplementary file 1 [file biomedicines-10-00336-s001.zip › biomedicines-1559051-supplementary.pdf]

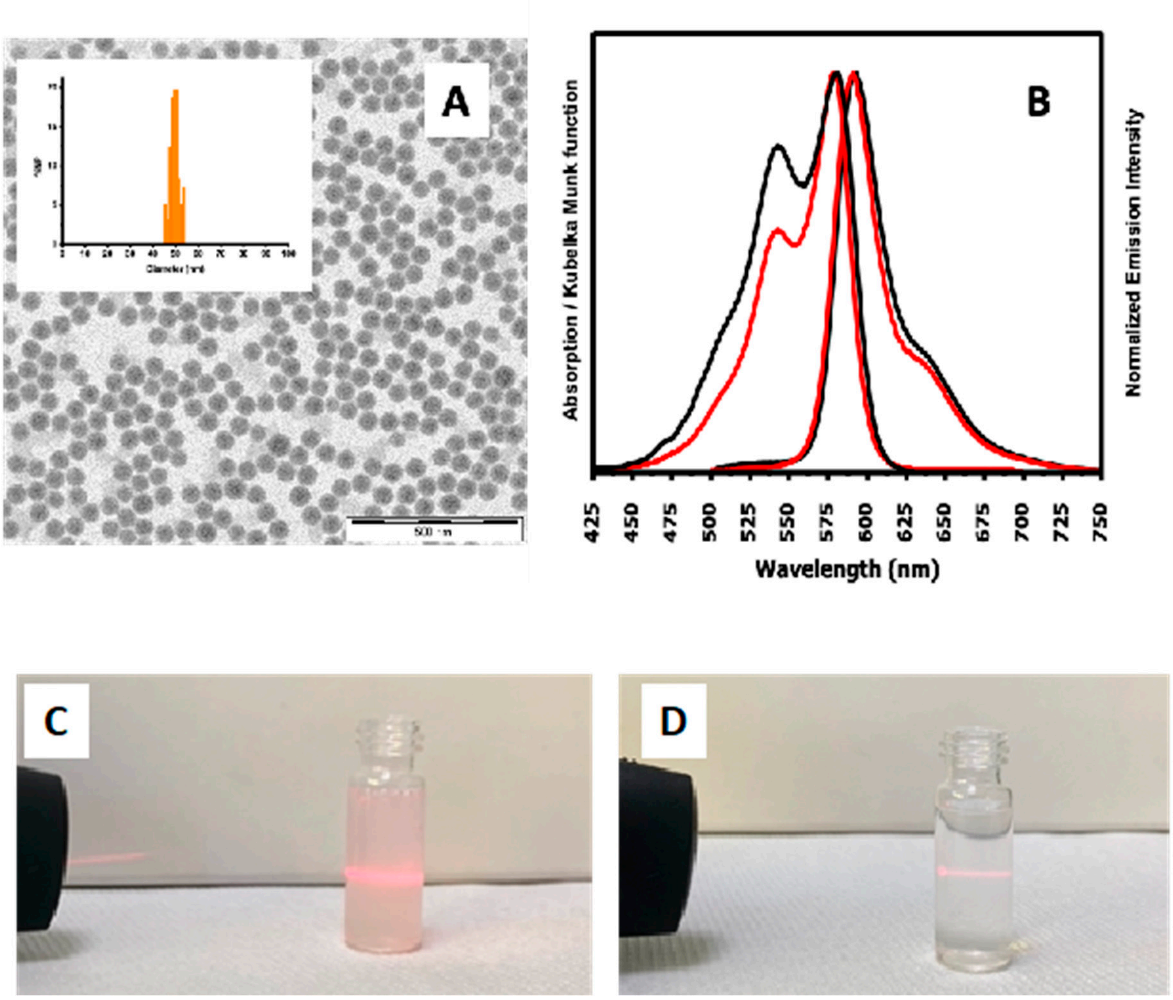

Figure S1: Production of SiO_2-NPs.

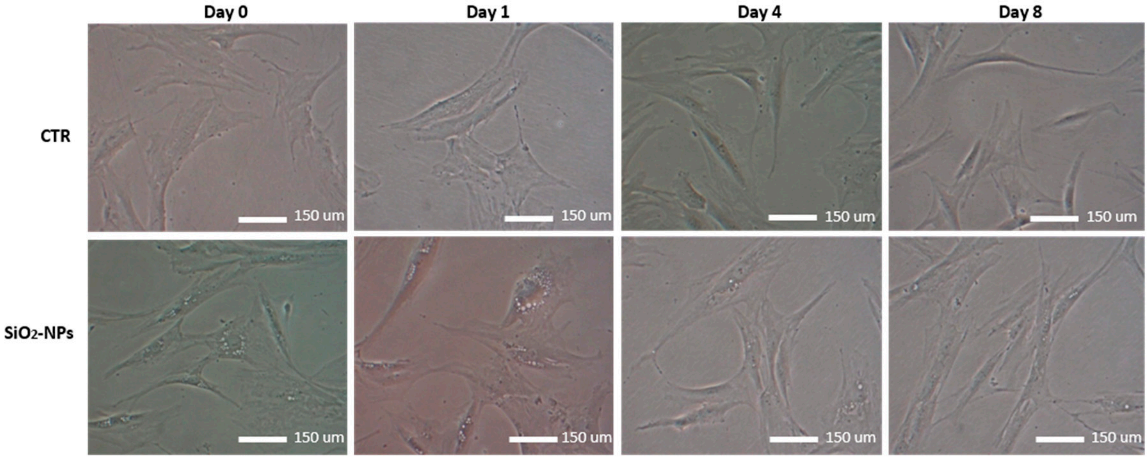

Figure S2: Bright field microscopy of SiO_2-NP-treated hMSCs.

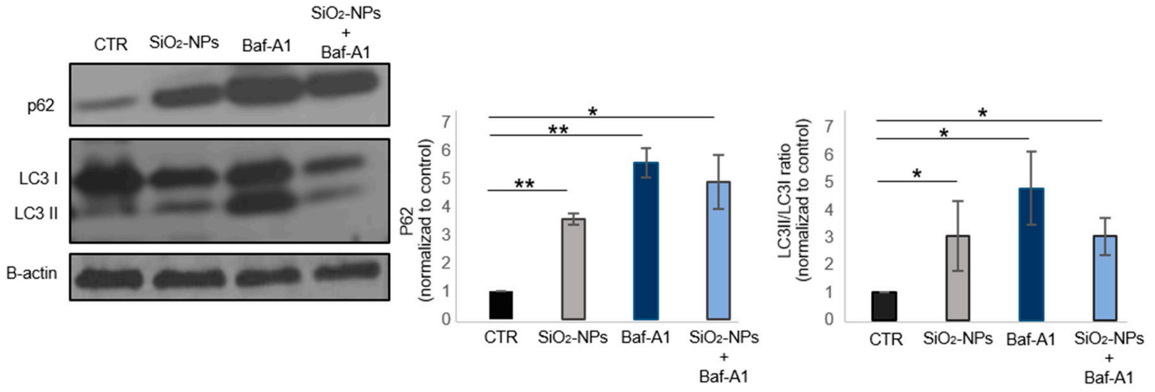

Figure S3: Autophagy marker analysis.

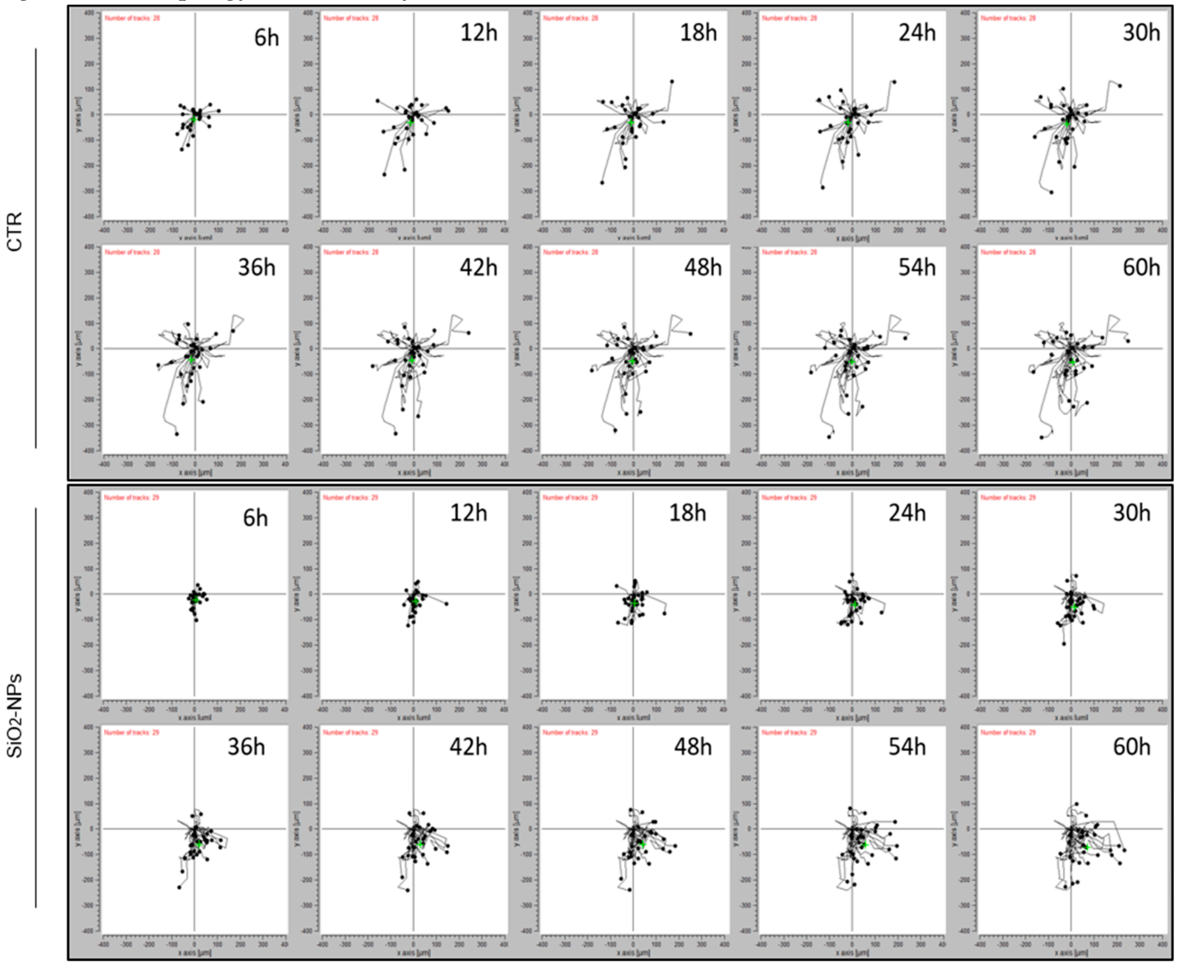

Figure S4: Trajectory plot of control and SiO_2-NP-treated hMSCs.
